# In the June 2014 issue

**DOI:** 10.1590/S1980-57642014DN82000001

**Published:** 2014

**Authors:** Sonia Maria Dozzi Brucki, Mônica Sanches Yassuda

**Affiliations:** Guest Editors

All of us are aware that cognitive performance is influenced by many factors. One of the most important of these is undoubtedly the initial educational experience of the individual. In low to middle-income countries, such as Brazil, schooling impacts the risk for dementia and hampers our ability to detect early cognitive changes. In this issue we are publishing four reviews and 11 original papers. These are largely dedicated to the difficulties, challenges and findings regarding education and cognition.

We were invited (Sonia MD Brucki and Mônica S Yassuda) by Dr. Ricardo Nitrini (Editor-in-Chief) to handle special articles about cognition, education and dementia. Our guests have submitted nine manuscripts, all of which were evaluated by peer reviewers, and the final result was very fruitful, conveying a wide variety of views and news in this field.

**Parra** provides us with a thorough review about challenges in evaluating cognition in developing countries and a discussion on innovative strategies. Most of the available assessment tools were created in developed countries, and although translated, sometimes these instruments are affected by cultural particularities.

**Pardo et al.** present a review of the Photo-Test (developed by the first author in Spain), which appears highly suited for evaluating cognitive impairment, especially in individuals with a low educational level.

**Paddick et al.** conducted an epidemiological study in Tanzania, where they observed a similar prevalence of dementia to rates documented in high-income countries (6.4%). The risk of having dementia was associated with having no formal education and also with illiteracy. The authors' findings highlighted the importance of dementia studies in developing countries to determine factors linked to dementia along with its health burden for low and middle-income societies.

**Yier et al.** studied relationships between literacy, age at dementia onset, and their confounding factors in demented patients from a University Hospital in Hyderabad (India). The authors showed that rural dwelling, stroke, and occupation can modify the relationship between education and dementia.

**Tripathi et al.** showed interesting findings for the effects of education, age and gender on neuropsychological functions among elderly Indians. They observed that measures of planning and working memory were among the areas most affected by education.

**Guimarães et al.** described factors associated with cognitive impairment and dementia among illiterate elderly persons in a sample from the Pietà epidemiological study in Minas Gerais (Brazil). This study revealed an association between vascular risk factors and impaired cognition, but not for other comorbidities, previous occupation or socioeconomic levels.

**Branco et al.** investigated the influence of education and frequency of reading and writing on verbal and visuospatial executive functions. They evaluated healthy elders using executive tasks and observed that education had an important impact on the scores, but also that the cognitive stimulation provided by reading and writing generated additional performance gains.

**Nunes et al.** presented interesting results from a neuropsychological stimulation program for elders from an elderly day care center in Portugal. They included individuals with low educational level and described results from a community-based perspective of action.

**Santos et al.** studied the quality of life in elderly persons and observed that those on literacy programs for adults had fewer depressive symptoms and an average quality of life.

Other manuscripts were included in this issue exploring a number of pertinent issues.

**Camozzato et al.** described interesting findings about mortality and successful aging in a cohort of older individuals from the PALA study in Porto Alegre (Brazil). They observed that mortality was greater among individuals with normal aging than among those with successful aging.

**Rosanti et al.** performed a one-year follow-up of elderly women divided into sedentary and active groups. They assessed participants at baseline and at the end of this period and noted that the active group had better performance on general cognitive function tests, especially for memory and praxis.

**Trés and Brucki** presented a systematic review on visuospatial processing, evaluating hierarchic processes involved in vision, attention and the relationships between dorsal and ventral pathways in these processes.

**Oliveira and Brucki** carried out an interesting review regarding computerized cognitive tests for evaluating patients with mild cognitive impairment and Alzheimer's disease. This represents a fertile field for present and future research.

**Oliveira et al.** also reviewed computerized tests for assessing the cognitive impact of interventions by describing the populations involved, limitations and advantages of this kind of task.

**Carezzato et al.** conducted a review on instruments for evaluating pain in severely demented patients. Detection of pain in these patients is a challenging task, particularly among individuals that can no longer verbalize their feelings.

**Sonia Maria Dozzi Brucki** **Mônica Sanches Yassuda** Guest Editor

